# Event-Related Potentials in Parkinson's Disease Patients with Visual Hallucination

**DOI:** 10.1155/2016/1863508

**Published:** 2016-12-08

**Authors:** Yang-Pei Chang, Yuan-Han Yang, Chiou-Lian Lai, Li-Min Liou

**Affiliations:** ^1^Department of Neurology, Kaohsiung Municipal Ta-Tung Hospital, Kaohsiung Medical University, Kaohsiung, Taiwan; ^2^Department of Neurology, Faculty of Medicine, College of Medicine, Kaohsiung Medical University, Kaohsiung, Taiwan; ^3^Department of Neurology, Kaohsiung Medical University Hospital, Kaohsiung, Taiwan

## Abstract

Using neuropsychological investigation and visual event-related potentials (ERPs), we aimed to compare the ERPs and cognitive function of nondemented Parkinson's disease (PD) patients with and without visual hallucinations (VHs) and of control subjects. We recruited 12 PD patients with VHs (PD-H), 23 PD patients without VHs (PD-NH), and 18 age-matched controls. All subjects underwent comprehensive neuropsychological assessment and visual ERPs measurement. A visual odd-ball paradigm with two different fixed interstimulus intervals (ISI) (1600 ms and 5000 ms) elicited visual ERPs. The frontal test battery was used to assess attention, visual-spatial function, verbal fluency, memory, higher executive function, and motor programming. The PD-H patients had significant cognitive dysfunction in several domains, compared to the PD-NH patients and controls. The mean P3 latency with ISI of 1600 ms in PD-H patients was significantly longer than that in controls. Logistic regression disclosed UPDRS-on score and P3 latency as significant predictors of VH. Our findings suggest that nondemented PD-H patients have worse cognitive function and P3 measurements. The development of VHs in nondemented PD patients might be implicated in executive dysfunction with altered visual information processing.

## 1. Introduction

Visual hallucinations (VHs) and cognitive impairment, which are nonmotor symptoms of Parkinson's disease (PD), have been an intriguing issue in recent years [1]. It is crucial to screen mild cognitive impairment and dementia in PD patients because dementia may cause nursing home placement, increased burden for health care and caregiver, and higher mortality [2]. In the mid-stage of PD, VHs act as a clinical predictor of dementia [3, 4] and correlate with disease progression and decline in Mini-Mental State Examination (MMSE) scores [5, 6]. Recent hypotheses suggest that the development of VHs in PD may result from an imbalance of external and internal inputs and impaired reality monitoring, while cognitive impairment may play a role in reality monitoring [7, 8]. Cognitive correlation of VHs in PD patients is evident [9–12]. A one-year neuropsychological follow-up study reported that nondemented PD patients with VHs have faster decline of complex visual function and multiple cognitive domains than patients without VHs [13]. Previous studies have also reported worse attention and visuospatial function in PD patients with VHs [14, 15]. However, another 4-year longitudinal observatory study showed that VHs may be more associated with longer disease duration, increased functional impairment, and premorbid psychiatry illness rather than cognitive impairment [16]. Accumulating evidence has demonstrated that cognitive dysfunction may contribute to the occurrence of VH symptoms of PD patients in nondemented PD patients with VHs regardless of the side effect of dopaminergic medication [17, 18]. Indeed, recent study using functional MRI technique suggests desynchronization between aberrant frontal circuit and posterior cortical areas during active visual hallucinations [19].

Event-related potential (ERP) is a developed sensitive and noninvasive tool to detect cognitive dysfunction in patients with mild cognitive impairment and dementia [20–22]. Early components of ERP (N1 and P2) are considered exogenous sensory components that have been associated with attention and sensory processing. The N2 component reflects an early detection of cognitive ability, such as target discrimination. P3 is a positive shift when a subject detects an informative task-relevant stimulus [23, 24]. While some studies have supported the correlation between ERP abnormality and cognitive impairment in PD patients with dementia, the role of ERP in nondemented PD patients is not confirmed [25–31]. One study found visual cognitive impairment and prolonged visual P3 latency especially in patients with PD dementia with hallucinations [32].

Since ERP may be a sensitive tool in the detection of cognitive impairment in PD in the absence of clinical dementia and VH is a potentially premonitory symptom of dementia in PD patients, it may be interesting to explore the ERP abnormality in nondemented PD patient with VHs. In the literature, few studies focused on the topic. We aim to assess the visual ERP and neuropsychological assessments in nondemented PD patients with (PD-H) and without VH (PD-NH) and healthy controls and find the linkage.

## 2. Materials and Methods

### 2.1. Participants

This study was conducted at the Kaohsiung Medical University Hospital (KMUH), a tertiary referral center in Southern Taiwan. The KMUH institution review board approved all procedures and written informed consent was obtained from study participants. The control subjects were recruited from volunteer in nearby community college. All PD participants had a presumptive clinical diagnosis of PD according to UKPD Brain Bank criteria. Individuals were inquired carefully and were assigned to groups according to whether they had experienced VHs in the past one year. No patient in the population sampled had a clinical diagnosis of either Alzheimer's disease or Lewy body dementia. Patients were excluded if Mini-Mental State Examination (MMSE) is less than 25. Patients with eye disease or migraine or other conditions like concurrent stroke, delirium, delusions, multiple sclerosis, and psychiatric illness or those under neuroleptics treatment were all excluded. Duration of illness and medication were recorded and stage of illness was scored according to the Hoehn and Yahr scale and United Parkinson's Disease Rating Scale (UPDRS) during “on” state. PD patients take neuropsychological assessment and event-related potential during “on” state after regular oral medications.

### 2.2. Neuropsychological Assessment

The neuropsychological assessment focusing on frontal lobe function [21] includes Mini-Mental State Examination (MMSE), Digit Span (Wechsler Adult Intelligence Scale-Revision (WAIS-R)), Digit Symbol (WAIS-R), Stroop Test, and Trail Making Tests (parts A and B) to assess attention and concentration; Block Design (WAIS-R) and Rey-Osterrieth Complex Figure Test-Copy to evaluate visuoconstructional ability; word list generation (Controlled Oral Word Association; Category Fluency Test) to assess semantic verbal fluency; word list learning-recall and Rey-Osterrieth Complex Figure Test-Recall to evaluate memory; Wisconsin Card Sorting Test-Modified (WCST), Design Fluency (Five-Point Test), and Similarity (WAIS-R) to assess higher executive function; Luria's Hand Sequence to evaluate motor programming function.

### 2.3. Event-Related Potentials Measurements

A visual ‘‘odd-ball” stimulus paradigm (NeuroStim, NeuroScan, Inc.) was used to elicit visual event-related potentials, and an electroencephalograph (EEG) was recorded using Ag/AgCl electrodes placed at 5 scalp locations (FPz, Fz, Cz, Pz, and Oz), based on the 10–20 system. All were referenced to linked earlobes. The electrode impedance was kept below 5 kΩ. The EEG was amplified (band pass, 0.01–40 Hz) by a SynAmps amplifier (NeuroScan, Inc.), and continuous EEG records were kept for further offline analysis at a sampling rate of 256 Hz. The averaging epoch was 1024 ms, including 200 ms of prestimulus baseline [21, 22].

The subjects sat in a comfortable chair in a sound-attenuated room with dim lighting 100 cm in front of a 19-inch LCD computer screen. Stimuli were presented in the central of the screen. The stimuli consisted of two neutral pictures from the NeuroScan template on a dark ground. The participants were asked to centrally fixate throughout the recording. We adopted a visual odd-ball task, with a target stimulus and a nontarget stimulus. Mistrials including eyeball movement artifacts were excluded from the offline analysis.

Stimuli were presented randomly with the probability of 20% target stimulus and 80% nontarget stimuli. In each block a total of 250 stimuli (50 targets, 200 nontargets) were presented for 100 ms and interstimulus interval (ISI) of 1600 ms and 5000 ms. The experiment consisted of 4 blocks (2 blocks with ISI of 1600 ms and 2 blocks with ISI of 5000 ms). Participants performed a brief training session to ensure they were able to detect the target accurately. During the examination, participants were asked to press the button as quickly as possible when they saw the target. Reaction time was measured relative to target onset for correct trials, while accuracy was measured as the percentage of correct responses out of all responses to the target stimulus. Individual trials with eye blink artifacts (more than 250 *μ*V of peak-to-peak amplitude), target trials for which the reaction time (RT) was more than 1.4 s, and nontarget trials with a response were all excluded from the averaging. Separate ERP averages were made for each trial type. For amplitudes analysis, the mean potential during the 200 ms period preceding the stimulus onset served as baseline. The N1, P2, N2, and P3 components at Pz recording were assessed for highest amplitude distribution. The latencies windows were N1 component as the maximum negativity between 75 and 160 ms, P2 component as the maximum positivity between 170 and 260 ms, N2 component as the maximum negativity between 190 and 360 ms, and P3 component as the maximum positivity between 250 and 500 ms.

### 2.4. Statistics

We performed statistical analysis with SPSS 12.0 package, and *p* < 0.05 was set to be statistically significant. We used two-tailed* t*-test for analyzing continuous data of disease characteristic of PD patients. We used analysis of covariate (ANCOVA) to determine the differences of neuropsychological test between groups after adjusting disease duration, duration of levodopa use, Hoehn and Yahr stage, Hamilton depression index, and the scores of UPDRS-III. For P3 latency and amplitude, we used ANCOVA to determine the significant difference after adjusting age, gender, disease duration, duration of levodopa use, and Hoehn and Yahr stage with Tukey method used for post hoc analysis. One-way repeated measures analysis of variance (ANOVA) was used to explore the effect of ISI on P3 latency and amplitude. Pearson correlation coefficient was calculated to explore the relationship between neuropsychological function and N1, P2, N2, and P3 latency and amplitude at Pz recording. ERP components at Fz and Cz recordings were not analyzed because of artifacts. For our intent in analyzing the predictive risk factors of VH, logistic regression with the existence or absence of VHs as dependent variable was performed in four different models with different confounding factors. To clarify the role of UPDRS-on score in the development of VH, we adjusted age and gender in model 1, while we adjusted age, gender, and three cognitive domains in model 3. To further observe the role of P3 latency in the development of VH, age and gender and UPDRS-on score were adjusted in model 2, while age and gender, UPDRS-on score, and three cognitive domains were adjusted in model 4. The chosen cognitive domains (Trail Making Tests, R-O copy, or Luria Hand Sequence) were significantly different between PD-H and PD-NH patients. According to WAIS-III Chinese version, the cut-off values of three cognitive domains were chosen and were transformed to dichotomy dummy variable for logistic regression.

## 3. Results

### 3.1. Demographic Data

Twelve PD-H patients, twenty-three PD-NH patients, and eighteen healthy control subjects were recruited in this study (Table 1). The mean age, education level, and MMSE did not differ significantly between these three groups, while there were significant differences between the PD patients with and without visual hallucinations with regard to disease duration, duration of levodopa use, Hoehn and Yahr stage, and the scores of UPDRS-III. We also found significant difference in Hamilton depression index in PD-H or PD-NH patients when comparing with normal controls.

### 3.2. Neuropsychological Assessment

The data of neuropsychological investigations in all participants are shown in Table 2. There were multiple domains of frontal dysfunction in PD patients, especially in PD-H patients. The PD-H patients performed significantly worse than normal controls at Trail Making Test, R-O copy, Wisconsin Card Sorting Test, and Luria Hand Sequence. Moreover, when comparing PD-H patients to PD-NH patients, significantly lower scores were found in the former group at Trail Making Test, R-O copy, Wisconsin Card Sorting Test, and Luria's Hand Sequence. When comparing to normal controls, PD-NH patients performed significantly worse in Wisconsin Card Sorting Test.

### 3.3. Visual ERP Data

For the highest amplitude distribution, the N1, P2, N2, and P3 components with two different ISI at Pz are outlined in Table 3. There was no significant difference between PD-H patients, PD-NH patients, and controls, regardless of different ISI (1600 ms and 5000 ms). However, the mean latency of P3 with ISI of 1600 ms in PD-H patients revealed significant prolongation when comparing with that in controls. The mean reaction time and error rate of PD-H patients, PD-NH patients, and controls revealed no significant difference.

We also assessed the effect of ISI on P3 latencies, P3 amplitude, and reaction time at Pz (Table 3) using one-way repeated measure ANOVA in PD-H patients, PD-NH patients, and controls. P3 latency was significantly prolonged at 5000 ms ISI compared to 1600 ms ISI in PD-NH patients and control (control, *F* = 19.289, *p* = 0.003; PD-NH, *F* = 5.391, *p* = 0.04), while PD-H patients did not show significant difference (*F* = 0.025, *p* = 0.879). P3 amplitude showed unremarkable difference in three groups when comparing 1600 ms ISI to 5000 ms ISI (PD-H, *F* = 0.324, *p* = 0.585; PD-NH, *F* = 2.987, *p* = 0.112; control, *F* = 1.031, *p* = 0.344). Reaction time was significantly prolonged at 5000 ms ISI compared to 1600 ms ISI in PD-NH and PD-H patients (PD-NH, *F* = 0.359, *p* < 0.001; PD-H, *F* = 13.059, *p* = 0.005), while there was no significant difference in controls (*F* = 2.831, *p* = 0.111).

### 3.4. The Correlations of Frontal Function and ERPs in PD-H Patients

The domains of frontal function in PD-H patients were analyzed for their correlation with measures of N1, P2, N2, and P3 at Pz lead. Pearson's* r* values of correlation between P3 latency, P3 amplitude, and neuropsychological scores are shown in supplementary Table 1 (in Supplementary Material available online at http://dx.doi.org/10.1155/2016/1863508). For higher executive function (Similarities, Wisconsin Card Sorting Test), attention (Trail Making Test-type B, Digit Span), visuoconstructional ability (Digit Span, Rey-Osterrieth Complex Figure copy test), verbal fluency (word list generation), and memory (Rey-Osterrieth Complex Figure recall test), there were significant correlations for P3. However, other earlier components of N1, P2, and N2 correlations with cognitive measures were not significantly evident (data not shown).

 Supplementary Table 2 summarizes the odds ratio of binary logistic regression for UPDRS-on score and P3 latency in different models. Overall, the results showed that increase of UPDRS-on scores in PD patients was associated with significantly increased risk of VH in four different models. After adjusting age, gender, and UPDRS-on scores, model 2 disclosed that one millisecond increase of P3 latency in PD patients was in line with 6%  (*p* = 0.046) higher risk of having VH. By contrast, model 3 showed that there was nonsignificant trend where poor performance of Trail Making Tests, R-O copy, or Luria Hand Sequence was more likely to have VH.

## 4. Discussion

Our study showed that nondemented PD patients with VHs had worse cognitive function than those without VHs and age-matched controls. In addition to UPDRS scores, the latency of visual P3 was associated with VH after statistically adjusting the possible confounding factors and also correlated with cognitive impairment in PD patients. In accordance with previous studies using neuropsychological assessment or functional MRI [15–18, 33, 34], our finding suggests that frontal dysfunction may play a role in the development of VH in nondemented PD patients.

The term of P300 is composed of mainly two distinct subcomponents, P3a and P3b. Although the precise functional origin of P300 induced by visual stimuli is controversial, visual P3b represents parietal cortical distribution reflecting the top-down allocation of attention resources to relevant stimuli [35–37]. As we measured our visual P3 latency as P3b, our P3 latency may reflect the top-down attribution of visual processing.

In the present study, P3 latency with ISI of 1600 ms in PD-H patients was significantly longer than control and associated with VH after adjustment of confounding factors. As P3 latency of ERPs increases in line with cognitive decline in Lewy body dementia patients and demented PD patients with VHs [29, 32, 38], our finding implies that visual cognitive functions are particularly impaired in nondemented PD patients with visual hallucinations. It is accepted that VHs in PD could be related to central cholinergic dysfunction in pedunculopontine nucleus [33, 39]. On the basis of indirect pharmacological evidence, P3 ERPs in Alzheimer's disease could reflect central cholinergic function [40, 41]. Hence, a possible explanation for our findings might be that nondemented PD patients with VHs might have more dysfunction over the frontobasal cholinergic pathways. In addition, visual ERP of fixed ISI with 1600 ms might be an auxiliary tool to detect cognitive dysfunction in nondemented PD.

There are several theoretical models implicated in the development of VHs in PD, and integrative approach may be needed to explore sensory, attention, and cognitive deficits [42]. Functional MRI during active VHs showed desynchronization between frontal and posterior cortical areas involved in visual processing [19], while Shine et al. suggest that decreased attentional network activity and increased primary visual system connectivity with default mode network may contribute to the development of VHs [43]. Our PD-H patient also showed significant deficits in tests about attention, visuoconstructional ability, executive function, and motor programming when comparing to PD-NH patients and control. However, latencies and amplitude of N1 ERP or P2 ERP, which may be more correlated with attentional network in brain, did not show significant differences between groups.

There were several limitations in our study. First, we collect PD patients from university-based hospital and the collection bias cannot be completely excluded. Secondly, visual ERP may be affected by excessive eyelid blinking related to blepharospasm, which is common in PD [44]. We did not exclude PD patients with blepharospasm in this study but the eyeball movement artifacts are excluded from the analysis. Thirdly, neuropsychological assessment may be affected by poor attention or decreased motor function in PD patients. We arranged the assessment in the morning and patients receive regular medications before the exam, but poor attention or motor fluctuation may still happen during the time-consuming tests.

## 5. Conclusion

We found that P3 ERPs measurements may be associated with visual hallucination and cognitive impairment in nondemented PD patients. Further longitudinal follow-up may be needed to confirm whether P3 ERP measurements and visual hallucinations might predict the development of dementia in PD patients

## Supplementary Material

The supplementary material consists of (1) correlation between P3 measurement and neuropsychological scores. (2) four models evaluating the odds ratio of UPDRS-on score and P3 latency of development of VH in PD.

## Figures and Tables

**Table 1:** Characteristics in PD-H, PD-NH, and controls.

	PD-H	PD-NH	Controls	*p*
	*n* = 12	*n* = 23		
Age^1^, years	67.79 ± 7.93	66.36 ± 9.68	68.29 ± 6.83	0.753
Women^1^, *n* (%)	5 (36)	7 (28)	10 (59)	0.107
Education^1^, y	9.27 ± 6.65	10.88 ± 4.16	12.16 ± 3.32	0.245
Disease duration^2^, y	**11.73 ± 6.41**	6.20 ± 4.86	n/a	**0.007**
Duration of levodopa^2^, y	**8.44 ± 5.78**	3.04 ± 3.57	n/a	**0.004**
H&Y^2^	**2.65 ± 0.89**	1.52 ± 0.65	n/a	**<0.001**
MMSE^1^ (score)	27.73 ± 2.20	27.58 ± 1.36	28.41 ± 1.37	0.472
HDI^1^ (score)	**5.00 ± 4.67**	4.125 ± 4.11^*∗*^	0.25 ± 0.79	**<0.001**

UPDRS-III motor^2^ (score)	**27.92 ± 13.00**	14.20 ± 8.42	n/a	**<0.001**
Levodopa-equivalent dose	863.8 ± 390.6	311.2 ± 160.5	n/a	0.08

PD-H: PD patients with visual hallucinations; PD-NH: PD patients without visual hallucinations; H&Y: Hoehn and Yahr stage; HDI: Hamilton depression index; UPDRS: Unified Parkinson's Disease Rating Scale; n/a: not available.

*p*: *p* value, *p* < 0.05; by ^1^ANOVA ^2^ *t*-test.

**Table 2:** Comparison of frontal test battery in PD patients and controls.

Demographic data	PD-H	PD-NH	Controls	*p*
mean ± SD	*n* = 12	*n* = 23	*n* = 18	
Attention				
Stroop Test (errors)	7.92 ± 9.09	7.33 ± 8.45	3.41 ± 5.19	0.151
TMT-A (s)	122.21**** ± ****87.47^ab^	70.84 ± 41.47	52.76 ± 30.27	**0.018**
TMT-B (s)	241.21 ± 121.73	134.00 ± 106.8	124.76 ± 84.81	0.264
Digit Span	15.64 ± 4.24	15.88 ± 2.76	17.27 ± 4.54	0.198
Visual-constructional ability				
Block Design (score)	7.21 ± 4.16	12.08 ± 4.05	11.50 ± 3.78	0.255
R–O-copy (score)	25.93 ± 10.17^ab^	32.08 ± 6.55	33.47 ± 1.91	**<0.001**
Verbal fluency				
Word list generation	41.00 ± 14.81	44.21 ± 11.29	46.47 ± 8.68	0.122
Memory				
Wordlist learning recall (number)	17.21 ± 3.66	19.19 ± 3.76	22.29 ± 1.45	0.301
R–O-recall (score)	7.71 ± 6.76	11.96 ± 9.19	13.00 ± 8.32	0.549
Higher executive function				
Similarities (score)	9.29 ± 6.08	10.12 ± 7.11	13.53 ± 4.58	0.760
Five-Point Test (correct number)	2.79 ± 2.46	4.57 ± 2.92	5.35 ± 2.52	0.485
WCST-category (number)	5.21 ± 2.12	6.00 ± 1.71	6.24 ± 2.31	0.697
WCST-PN/total errors (%)	63.00 ± 32.91^b^	72.80 ± 31.40^c^	25.29 ± 20.95	**0.027**
Motor programming				
Luria's Hand Sequence (score)	1.29 ± 1.07^ab^	2.23 ± 1.14	2.12 ± 0.68	**0.019**

PD-H: PD patients with visual hallucinations; PD-NH: PD patients without visual hallucinations; TMT: Trail Making Test; R–O Complex Figure Test: Rey-Osterrieth Complex Figure Test; WCST: Wisconsin Card Sorting Test.

*p*: *p* value, by one-way analysis of covariance (ANCOVA) with age, gender, and education as covariates.

Post hoc analysis with Tukey method (^a^PD-H versus PD-NH, ^b^PD-H versus controls, and ^c^PD-NH versus controls).

**Table 3:** Comparisons of latencies and amplitude at Pz in visual ERPs of PD-H, PD-NH, and controls.

	PD-H	PD-NH	Controls	*p*
	*n* = 12	*n* = 23	*n* = 18	
Amplitude, uV				
ISI = 1600 ms				
N1	−0.59 ± 3.19	−2.48 ± 4.75	−2.57 ± 5.18	0.479
P2	7.05 ± 3.81	10.87 ± 6.75	7.18 ± 5.73	0.164
N2	−0.07 ± 5.42	0.95 ± 7.77	−1.33 ± 6.04	0.388
P3	12.19 ± 4.74	14.14 ± 9.76	11.62 ± 7.86	0.934
ISI = 5000 ms				
N1	−1.44 ± 3.42	−2.02 ± 1.81	−2.26 ± 4.25	0.351
P2	6.40 ± 3.40	5.83 ± 3.81	6.18 ± 4.89	0.831
N2	−1.57 ± 5.08	−1.24 ± 4.39	−1.48 ± 4.32	0.836
P3	11.18 ± 5.21	12.38 ± 8.33	13.34 ± 6.50	0.831
Latency, ms				
ISI = 1600 ms				
N1	140.29 ± 13.03	139.43 ± 21.83	133.73 ± 15.34	0.585
P2	183.67 ± 15.40	185.57 ± 22.67	176.60 ± 8.09	0.643
N2	277.11 ± 33.45	262.85 ± 18.26	263.92 ± 18.67	0.288
P3	396.44**** ± 28.19^a^	366.57 ± 21.58	359.89 ± 27.89	**0.005**
ISI = 5000 ms				
N1	152.86 ± 22.68	131.17 ± 20.25	135.63 ± 13.19	0.594
P2	215.14 ± 26.02	191.13 ± 29.20	197.11 ± 18.28	0.391
N2	282.75 ± 34.45	278.00 ± 22.94	265.17 ± 22.41	0.498
P3	397.38 ± 18.78	395.89 **** ± 28.29^b^	404.44**** ± ****39.88^b^	0.827
RT, ms				
ISI = 1600 ms	434.39 ± 71.49	395.65 ± 77.05	382.70 ± 52.97	0.145
ISI = 5000 ms	480.81 **** ± 88.60^b^	450.64**** ±**** 94.03^b^	421.29 ± 121.03^b^	0.321
Error rate				
ISI = 1600 ms	0.03 ± 0.02	0.04 ± 0.05	0.19 ± 0.02	0.178
ISI = 5000 ms	0.03 ± 0.04	0.02 ± 0.02	0.02 ± 0.02	0.552

PD-H: PD patients with visual hallucinations; PD-NH: PD patients without visual hallucinations; RT: reaction time. Values are expressed as mean ± SD.

*p*: *p* value, by one-way analysis of covariance (ANCOVA) with age, gender as covariate for between-group comparison and by one-way repeated measures analysis of variance (ANOVA) for within-group comparison.

^a^ *p* < 0.05, PD-H versus control, Tukey method for post hoc analysis,

^b^ *p* < 0.05, ISI = 5000 ms versus 1600 ms, by paired *t*-test.
