# Supplementary material for: Event-Related Potentials in Parkinson's Disease Patients with Visual Hallucination
Source: Parkinsons Dis. 2016 Dec 8;2016:1863508. doi: 10.1155/2016/1863508 (PMC5178355; doi:10.1155/2016/1863508)
Supplement: Supplementary file 1 — The supplementary material consists of (1) correlation between P3 measurement and neuropsychological scores. (2) four models evaluating the odds ratio of UPDRS-on score and P3 latency of development of VH in PD. [file 1863508.f1.docx]

**Supplementary tables**

**Table 1. Correlation between frontal test battery and P3 measurements in ISI of 1600ms in PD-H**

|  | Fz |  | Cz |  | Pz |  |
| --- | --- | --- | --- | --- | --- | --- |
|  | L | A | L | A | L | A |
| **Attention** |  |  |  |  |  |  |
| Stroop Test (errors) | 0.284 | -0.244 | 0.262 | -0.240 | 0.235 | -0.140 |
| TMT-A (s) | 0.480 | -0.483 | 0.490 | -0.418 | 0.493 | -0.468 |
| TMT-B (s) | 0.518 | **-0.621*** | 0.513 | **-0.593*** | 0.512 | -0.574 |
| Digit Span (scale score) | -0.533 | **0.744*** | -0.510 | **0.805*** | -0.558 | **0.784*** |
| **Visuo-constructional ability** |  |  |  |  |  |  |
| Block Design (scale score) | **-0.597*** | **0.749*** | -0.565 | **0.682*** | -0.552 | **0.716*** |
| R–O-copy (score) | **-0.587*** | 0.487 | **-0.598*** | 0.427 | **-0.584*** | 0.448 |
| **Verbal fluency** |  |  |  |  |  |  |
| Word list generation (number) | -0.339 | **0.778*** | -0.306 | **0.863*** | -0.330 | **0.807*** |
| **Memory** |  |  |  |  |  |  |
| Word list learning-recall (number) | -0.092 | 0.235 | -0.093 | 0.203 | -0.071 | 0.214 |
| R–O-recall (score) | **-0.584*** | 0.495 | **-0.611*** | 0.448 | **-0.610*** | 0.465 |
| **Higher Executive function** |  |  |  |  |  |  |
| Similarities (scale score) | **-0.696*** | **0.750*** | **-0.658*** | **0.717*** | **-0.647*** | **0.729*** |
| Five-Point Test (correct number) | -0.292 | 0.299 | -0.292 | 0.367 | -0.342 | 0.420 |
| WCST-category (number) | -0.149 | 0.587 | 0.134 | **0.603*** | 0.140 | -0.517 |
| WCST-PN/total error (%) | 0.313 | **-0.633*** | 0.302 | **0.691*** | 0.331 | **-0.774*** |
| **Motor programming** |  |  |  |  |  |  |
| Luria’s Hand Sequence (score) | 0.123 | 0.162 | 0.151 | 0.178 | 0.135 | 0.261 |

PD-H, PD patients with visual hallucinations; PD-NH, PD patients without visual hallucinations; TMT: Trail Making Test; R–O Complex Figure Test: Rey–Osterrieth Complex Figure Test; WCST: Wisconsin Card Sorting Test; PN: Perseveration error; L: latency; A: amplitude.

* p<0.05 by Pearson product–moment correlation

**Supplementary tables**

**Table 2. The effect of associated factors on visual hallucinations in PD**

|  |  | Model 1 | | | Model 2 | | | Model 3 | | | Model 4 | | |
| --- | --- | --- | --- | --- | --- | --- | --- | --- | --- | --- | --- | --- | --- |
|  | Variable | OR | 95%CI | P | OR | 95%CI | P | OR | 95%CI | P | OR | 95%CI | P |
| Age | <65 | 1.00 |  |  | 1.00 |  |  | 1.00 |  |  | 1.00 |  |  |
|  | ≧65 | 0.946 | 0.36-2.48 |  | 0.725 | 0.24-2.20 |  | 0.732 | 0.24-2.22 |  | 0.547 | 0.15-2.01 |  |
| Gender | Female | 1.00 |  |  | 1.00 |  |  | 1.00 |  |  | 1.00 |  |  |
|  | Male | 1.34 | 0.217-8.291 |  | 2.49 | 0.28-22.94 |  | 1.26 | 0.17-9.64 |  | 2.74 | 0.18-42.66 |  |
| UPDRS-on |  | **1.15** | 1.04-1.26 | **0.005** | **1.16** | 1.03-1.31 | **0.013** | **1.16** | 1.03-1.30 | **0.018** | **1.18** | 1.03-1.36 | **0.021** |
| P3 latency |  |  |  |  | **1.05** | 1.01-1.10 | **0.024** |  |  |  | **1.06** | 1.01-1.11 | **0.046** |
| TMT A | ≦76 |  |  |  |  |  |  | 1.00 |  |  | 1.00 |  |  |
|  | >76 |  |  |  |  |  |  | 1.75 | 0.62-11.69 |  | 1.31 | 0.13-12.97 |  |
| R-O copy | ≧31 |  |  |  |  |  |  | 1.00 |  |  | 1.00 |  |  |
|  | <31 |  |  |  |  |  |  | 2.22 | 0.30-16.62 |  | 0.99 | 0.08-12.23 |  |
| Luria HS | ≧1 |  |  |  |  |  |  | 1.00 |  |  | 1.00 |  |  |
|  | <1 |  |  |  |  |  |  | 11.00 | 0.40-307.17 |  | 10.99 | 0.35-346.79 |  |

OR, Odds Ratio; UPDRS, Unified Parkinson’s disease Rating scale; TMT: Trail Making Test; R–O Complex Figure Test: Rey–Osterrieth Complex Figure Test; Luria HS, Luria's hand sequence

p, p-value, by binary logistic regression
